# Modern Treatment of Burns

**Published:** 1919-05-24

**Authors:** 


### MODERN TREATMENT OF BURNS.

The use of paraffin preparations represents but a part of the advance in the treatment of burns, and forms a technical rather than therapeutic development. For application of such covering to the damaged tissues no antiseptic action is claimed, but the degree by which pain is relieved is remarkable, and in itself gives the method considerable right to general adoption. Apart from forming a protective film-like covering replacing the epidermis, the paraffin apparently acts as a purifying factor by tending to collect in its substance much of the oily contaminating dirt to which many such wounds have been exposed. In parallel with modern tendencies in all wound treatment, the method aims to shield and encourage the natural processes of tissue repair without artificial attack upon bacterial infection. The principle is by no means new, but the simplicity of such dressings, the ease with which the materials can be carried and the patient's comfort constitute a great improvement upon any previous methods.

There are by now a number of preparations on the market, but perhaps the best known is Ambrine, originally introduced by Dr. Barthe de Sandfort in 1915-1916, and understood to consist essentially of paraffin with the addition of oleum succini. These materials melt at about 120-124° F., and may be applied with a brush, as suggested in the directions supplied by the makers, but preferably, as experienced in service hospitals, by means of a simple metal spray. The Naval Medical Service uses an efficient form, in which ambrine may be melted up either in a water-bath or over a spirit-flame, and the apparatus used repeatedly, as the contained material may be liquefied many times without losing its essential properties. The whole provides a most convenient means of application in either hospital or mobile units.

As in all such simple methods, there are several equally simple essentials to success. The wounds must be thoroughly cleansed in sterile water, the addition of even dilute antiseptics being considered unnecessary. Burnt surfaces must be dried as thoroughly as possible by touching, not rubbing, with sterile wads of cotton wool, and with great care to avoid the production of oozing or exudation. In hospital a current of hot air is most useful for this purpose. Later, no bleeding from granulations is to be allowed—and in the spray itself care should be taken to have a dry container and allow no beads of water to enter the paraffin from the surrounding bath.

If this ideal technique be employed a perfect film is formed, and is both sterile, from the temperature at which it is employed, and airtight. Solidification is very rapid. Above the initial layer is placed a very thin sheet of absorbed cotton, also fixed in place by free application of the spray, and the whole is then covered with the usual cotton wool and bandage. A strong perfect layer of paraffin must always intervene between the fabric and the wound surface, otherwise sticking and ruin of the whole method will occur. Normally, however, the dressing may be removed easily by cutting with scissors through the whole cast, warmth as an air current or hot sterile swab aiding the process at any point where faulty application has caused slight adherence.

It is found that in extensive burns the dressing must be at first renewed every twenty-four hours, owing to the amount of exudation, but later the interval may often be doubled. Free serous exudation, or even a mild unpleasantness of odour, is not a contra-indication to paraffin, for in numbers of such cases granulation and the good growth of epithelium may be seen throughout. With obviously septic states it is, of course, another matter, but the paraffin method may be ultimately employed in a great number of these after the usual stage of surgical cleansing has been passed.

Advocates of such treatment claim that not only is healing accelerated, but that both scarring and ultimate contraction are markedly reduced. This fact in itself would constitute an invaluable advance, for, wonderful though recent plastic surgery has proved itself to be, the tremendous deformity often caused by burns under former conditions of healing would have set the surgeon a truly difficult task. Actually it would seem, after three years have elapsed and every opportunity to observe the wonderful results obtained in thousands of burns cases, that equal credit is due to such initial healing technique and to modern surgery, in not only reducing deformity and disfigurement, but, far more important, in the restoration of functional perfection.

